# New Players in the Infertility of a Mouse Model of Lysosomal Storage Disease: The Hypothalamus-Pituitary-Gonadal Axis

**DOI:** 10.3389/fendo.2013.00204

**Published:** 2014-01-06

**Authors:** Paola Piomboni, Laura Governini, Martina Gori, Erica Puggioni, Elvira Costantino-Ceccarini, Alice Luddi

**Affiliations:** ^1^Department of Molecular and Developmental Medicine, University of Siena, Siena, Italy

**Keywords:** spermatogenesis, Twitcher mouse, Krabbe disease, gene expression, hypothalamus-pituitary-gonadal axis

## Abstract

Mammalian spermatogenesis is a complex hormone-dependent developmental program where interactions between different cell types are finely regulated. Mouse models in which any of the sperm maturation steps are perturbed provide major insights into the molecular control of spermatogenesis. The Twitcher mouse is a model for the Krabbe disease, characterized by the deficiency of galactosylceramidase (GALC), a lysosomal enzyme that hydrolyzes the terminal galactose from galactosylceramide, a typical component of the myelin membrane. In addition, GALC catalyzes the hydrolysis of the terminal galactose from galactosyl-alkyl-acyl-glycerol, precursor of seminolipids, specifically expressed on the membrane of germ cells. Previous data reported by our group demonstrated that glycolipids play an important role in sperm maturation and differentiation. Moreover, we hypothesized that the severe impairment of the central nervous system that affects the Twitcher mouse could interfere with the hypothalamus-pituitary-gonadal axis function, contributing to infertility. To highlight this hypothesis we have determined, at molecular level, the potential variation in expression pattern of brain hormones involved in spermatogenesis regulation.

## Introduction

Infertility is a major medical problem worldwide. Male infertility affects 1 in 25 men in the Western world and is the cause of considerable social and financial burden (1).

Spermatogenesis is a complex series of events which collectively involve the coordinated expression of about 2300 different genes (2, 3). Given the complex cellular and molecular interactions that are involved in spermatogenesis, the whole process cannot be modeled *in vitro*. However, mouse models provide an attractive alternative since the great majority of the genes and processes involved in sperm production are conserved between mice and men, thus making mice excellent models of human infertility (4, 5).

It is known that spermatogenesis in mammals requires the action of a complex assortment of peptides and hormones each of which plays an important role in the normal functioning of the seminiferous epithelium (6, 7). The gonadotropin-releasing hormone (GnRH), secreted from the hypothalamus, stimulates the anterior pituitary to release follicle-stimulating hormone (FSH) and luteinizing hormone (LH). In turn, these two hormones regulate gametogenesis, hence the brain has a pivotal role in the control of spermatogenesis (8). LH stimulates the interstitial steroidogenic Leydig cells to produce testosterone, which has a local effect on interstitium and seminiferous tubules resulting in sperm production and maturation (9). FSH exerts its effect directly on the Sertoli cells whose direct contact with proliferating and differentiating germ cells within the seminiferous tubules makes them essential for providing both physical and nutritional support for spermatogenesis (10–12). Testosterone and estradiol, the latter converted through aromatase in the testis interstitium as well as in germ cells (13), are direct negative feedback modulators of GnRH, LH, and FSH (14).

Hence the maintenance of the proper crosstalk between the nervous system and the male gonads is mandatory for male fertility. This relationship becomes obvious if we take into account several unlinked autosomal mutations, which cause defects in both systems. Several studies on Lysosomal Storage Diseases (LSDs), genetic disorders caused by lysosomal enzyme deficiencies, demonstrate that lysosomal enzymes can elicit pleiotropic effects specifically on spermiogenesis (15, 16). In fact, in the knockout mice for the lysosomal enzymes sphingomyelinase α, H-hexosaminidase, or arylsulfatase A, both nervous and reproductive system are affected (17–19).

## Twitcher Mouse Spermatogenesis

**Figure 1:**
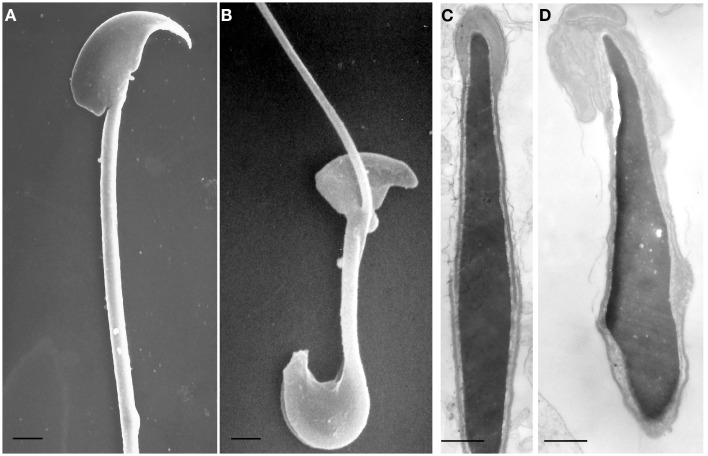
**Scanning (A,B) and transmission (C,D) electron microscopy micrographs of spermatozoa from wild type (A,C) and Twitcher (B,D) mouse, collected from vas deferens**. In **(A)**, head and tail of control mouse sperm have a normal morphology: the crescent-like shape of the head and the acrosomal profile are evident and the flagellum is well developed. The sperm from Twitcher mouse shows the typical hairpin morphology **(B)**. At transmission electron microscopy level, sperm from control mouse show a normal structure of both acrosome and nucleus, with a well condensed chromatin **(C)**. By contrast, the acrosome of the Twitcher sperm is aberrant and detached from the nucleus, the plasma membrane is also enlarged and redundant; the nuclear profile is irregular and the chromatin appears granular and uncondensed **(D)**. [**(A,B)**: bars = 2 μm; **(C,D)**: bars = 0,25 μm]. Modified from Ref. (23).

New insights come also from the Twitcher mouse, a naturally occurring model of Krabbe disease, characterized by deficiency of galactosylceramidase (GALC) (20, 21). GALC is a lysosomal enzyme that hydrolyzes the terminal galactose from galactosylceramide, a typical component of the myelin membrane, and from galactosyl-alkyl-acyl-glycerol (GalAAG), precursor of seminolipids, glycolipids expressed on the membrane of germ cells (22). We have previously demonstrated that GALC deficiency causes metabolic and structural abnormalities in the spermatozoa of the Twitcher mouse as consequence of a significant accumulation of undegraded GalAAG and minor alterations in the concentration of seminolipids (23). In comparison with sperm obtained from wild type mice (Figures 1A,C), the spermatozoa of the Twitcher mouse recovered from the cauda epididymis or vas deferens (Figures 1B,D) reveal significant structural defects affecting both head and tail. Scanning electron microscopy analysis shows an altered shape of the sperm head (Figure 1B), which appears reduced in size and devoid of the acrosomal profile. Often the tail appears coiled at the level of the cytoplasmic droplet causing an incorrect development of the flagellum and its cytoskeletal structures (Figure 1B).

At ultrastructural level, the most severe alterations are detected in the acrosomal complex (Figure 1D): the inner acrosomal membrane is completely detached from the nucleus, the acrosome is swollen, redundant, and folded over. Furthermore, the plasma membrane is also enlarged and redundant. The nuclear profile is irregular and the chromatin appears granular and less compact than in control sperms (Figure 1C). These morphological abnormalities, the significant accumulation of undegraded GalAAG and the minor alterations in the concentration of seminolipids, previously reported in Twitcher mice by our group, demonstrated the pleiotropic effect of the *GALC* gene suggesting its importance in the development and function of the male reproductive system and indicating in its deficiency the cause of infertility of the Twitcher males.

It is known that hormones play a key role in controlling spermatogenesis and, moreover, that neurological impairment is often associated to infertility as demonstrated in several neurological mouse mutants. We have, therefore, hypothesized that an unbalanced hormonal profile, owing to severe brain degeneration, could contribute to male infertility in the Twitcher mouse.

At testicular level, the Leydig and Sertoli cells are the target of pituitary hormones, such as LH and FSH. The close interaction between germ cells and somatic cells, present in testis, was demonstrated to be essential for correct spermatozoa differentiation. Any alteration in their morphology/metabolism would result in the impairment of this relationship.

Among the testicular interstitial cells, Leydig cells are very important in testis development since they produce testosterone, a steroid hormone with a pivotal role in the regulation of spermatogenesis. To evaluate potential Leydig cells dysfunction, a careful morphological investigation of the tubular tissue of Twitcher mouse was performed in 35 days old mice, when the spermatogenetic process is already completed.

**Figure 2:**
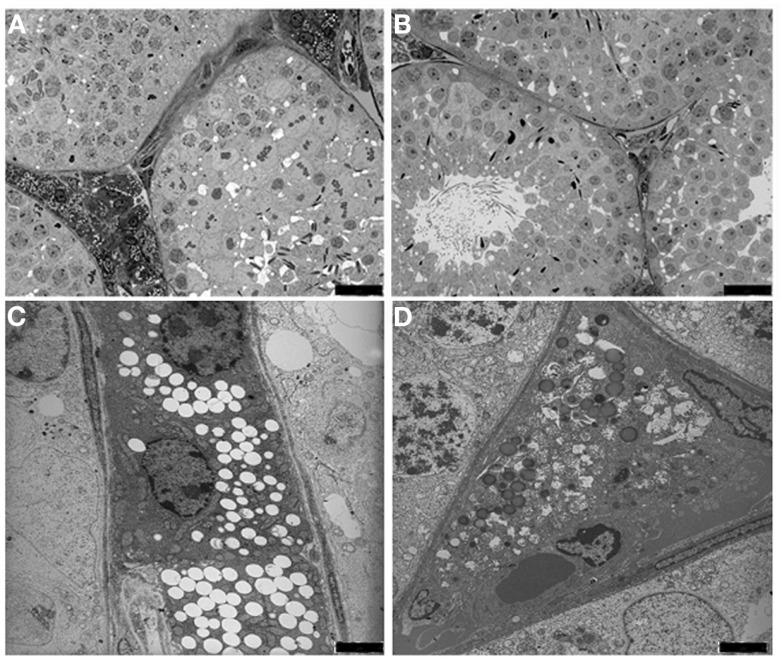
**Light microscopy (A,B) and transmission electron micrograph (C,D) of the interstitium from wild type (A,C) and Twitcher (B,D) testes**. In the peritubular interstitium of wild type mice numerous Leydig cells are presents and filled with lipids droplets and lysosomes, whereas in Twitcher mice the interstitial cells are strongly reduced in number and appear partially degenerated [**(A,B)**: bars = 25 μm;**(C,D)**: bars = 2 μm].

We observed, at light microscopy level, that Twitcher mouse tubules compared to age matched wild type were smaller in size and that the interstitial space was reduced allowing the tubular membranes to become adjacent (Figures 2A,B). These results indicate a loss not only of Leydig, but also of myoid cells.

At the ultrastructural level the Leydig cells of wild type mice were found in small clusters and most of them showed a normal ultrastructural pattern, with cell cytoplasm containing many lipid droplets (Figure 2C). Leydig cells from the Twitcher mice appeared to be degenerated showing a significant decrease in the number of lipid droplets (Figure 2D). Based on the established correlation between the amount of testosterone and the number of lipid droplets (24, 25), a reduction of its synthesis in Leydig cells can be hypothesized.

## Hypothalamic-Pituitary-Gonadal Axis

Gonadotropin-releasing hormone, secreted by hypothalamic neurons, is a key integrator between the neural and endocrine systems that stimulates the synthesis, storage, and secretion of gonadotropins by gonadotropic cells in the anterior pituitary. FSH and LH are the primary gonadotropins; in males, they stimulate testicular function through specific receptors (LH-R and FSH-R) expressed by Leydig and Sertoli cells, respectively. Thus, GnRH, FSH, and LH are the brain hormones that regulate testicular function and spermatogenesis. To establish if the hypothalamic-pituitary-gonadal axis is deregulated in the Twitcher mice, we have investigated by qRT-PCR the mRNA expression levels of genes encoding these hormones. The expression levels of the analyzed genes in wild type (*n* = 6) and Twitcher mice (*n* = 6) at PNDs 35, were normalized to the validated housekeeping gene eEF-2 (Eukaryotic elongation factor 2 kinase) (26) and referred to the wild type mouse (considered to be equal to 1).

Our results indicated that, at PND 35, GnRH expression is reduced by 70% in the Twitcher brain compared to wild type (*p* < 0.01). LH and FSH expression, were also significantly decreased (50 and 80% respectively, *p* < 0.05) in the Twitcher brain compared to aged matched wild type mouse.

Thus, gene expression analysis performed at brain level proved that hypothalamus and pituitary functions were affected.

## Conclusion and Open Questions

Since mammalian spermatogenesis is a complex hormone-dependent developmental program that ultimately give rise to spermatozoa, mouse models in which any of this step is perturbed have provided major insights into the molecular control of spermatogenesis.

The studies presented are the follow up of previous observations published by our group providing clues to the pleiotropic effect of the GALC gene and its importance in the development and function of the male reproductive system (23). The data that we have described demonstrate that the altered lipid metabolism, due to GALC deficiency, is not the only cause of male infertility. In addition, they support the hypothesis that the severe and progressive degeneration of the CNS affects the hypothalamus and hypophysis function, thus interfering with hypothalamus-pituitary-gonads axis. In fact, the GnRH produced by the hypothalamus mediates the secretion of the gonadotropin hormones FSH and LH by the hypophysis, that in turn regulate the testicular functions through their receptors (11, 27).

In conclusion, the data presented demonstrate that, in this mutant, the infertility may not be exclusively caused by the metabolic abnormalities in the sphingolipid pathway due to the GALC defect but, rather, to the severe involvement of the CNS that causes disruption of the hypothalamus-pituitary-gonadal axis.

Although further work is needed to fully clarify the complex interaction between brain and testis hormones, our data offer a new approach to study the spermatogenesis defects associated to CNS pathologies. Furthermore, the Twitcher mouse can be considered a model system for the study of hormone signaling orchestration between brain hormones with their testicular receptors.

## Conflict of Interest Statement

The authors declare that the research was conducted in the absence of any commercial or financial relationships that could be construed as a potential conflict of interest.
